# Spatiotemporal Distribution of Continuous Precipitation and Its Effect on Vegetation Cover in China over the Past 30 Years

**DOI:** 10.3390/plants15081198

**Published:** 2026-04-14

**Authors:** Hui Zhang, Shuangyuan Sun, Zihan Liao, Tianying Wang, Jinghan Xu, Peishan Ju, Jinyu Gu, Jiping Liu

**Affiliations:** 1 Geographical Science and Tourism College, Jilin Normal University, Siping 136000, China; zhanghui12878@163.com (H.Z.); lxy1871649349@163.com (Z.L.); w13756772134@163.com (T.W.); jinghan_xu1116@163.com (J.X.); jupeishan_lisa@163.com (P.J.); 13894081009@163.com (J.G.); 2 Northeast Institute of Geography and Agroecology, Chinese Academy of Sciences, Changchun 130033, China; sun15526963795@163.com

**Keywords:** continuous precipitation event, vegetation growth, spatial distribution, frequency, hydrology balance

## Abstract

Precipitation is a fundamental element in terrestrial water circulation and ecosystem hydrological balance. The occurrence of concentrated precipitation is closely linked to vegetation growth and soil fertility rather than accumulated or averaged precipitation. Despite its importance, the characteristics of continuous precipitation and its specific effects on vegetation cover remain uncertain. In this study, we formulated a new continuous precipitation index system, including $CP_{d}$ (continuous precipitation days); $ACP_{t}$ (annual continuous precipitation times); $CP_{a}$ (continuous precipitation amount); and FCP (frequency in different ranges of $ACP_{a}$). We utilized daily precipitation data from 467 meteorological stations across China, which were divided into eight vegetation type regions. We observed that the spatial distribution of continuous precipitation differed to varying degrees from accumulated precipitation. The national average of $MACP_{a}$ for a single event was 16.7 mm, ranging from 3.8 mm in the temperate desert region to 37.1 mm in the tropical monsoon forest and rainforest region. Similarly, the national average of $MCP_{d}$ ($MMCP_{d}$) for a single event was approximately 2.3 or 9 days. At the regional level, the tropical monsoon forest and rainforest region experienced the longest $MMCP_{d}$. Furthermore, the national average of $MACP_{t}$ occurrences for 1 year was 57.7 times, varying from 29.8 times in the temperate desert region to 77.9 times in the tropical monsoon forest and rainforest region. Vegetation responses to precipitation regimes exhibit significant regional heterogeneity across China. Our analysis reveals that $MACP_{t}$ and $MP_{a}$ show markedly positive correlations with vegetation growth. In subtropical monsoon climate zones, particularly the Yunnan–Guizhou Plateau and Qinling Mountains, $MACP_{t}$ demonstrates strong positive correlations (r = 0.6–1.0) with NDVI, where sustained rainfall provides stable moisture availability for vegetation. While a positive correlation between vegetation (NDVI) and mean annual consecutive precipitation is observed in some arid northern regions, in ecosystems such as the Loess Plateau (TG/TM), vegetation growth shows greater dependence on $MP_{a}$, highlighting the crucial role of total precipitation amount in water-limited ecosystems. Notably, extreme precipitation events display dual effects on vegetation dynamics. Prolonged heavy rainfall ($MMCP_{d}$/$MMCP_{a}$) exhibits significant negative impacts on NDVI (r = −1.0 to −0.6) in topographically complex regions, including the Hengduan Mountains and Yangtze River Basin (SE), likely due to induced soil erosion and waterlogging stress. Our findings underscore the importance of incorporating continuous precipitation indices to evaluate and forecast the influence of precipitation on ecosystem stability. This understanding is vital for developing informed conservation and management strategies to address current and future climate challenges.

## 1. Introduction

Precipitation plays a vital role in the regional water balance within terrestrial ecosystems and significantly influences a variety of ecological processes, contributing to the stability of ecosystems; for instance, it affects vegetation phenology and cover, as well as soil structure and fertility [1,2,3]. The effects of global warming have led to significant alterations in the surface water cycle, resulting in increased water transfer from the surface to the atmosphere. This amplification has intensified the frequency and severity of extreme weather phenomena. Rising temperatures have the potential to accelerate the melting of polar snow and high-altitude glaciers, thereby contributing to sea level elevation [4,5,6,7]. The Intergovernmental Panel on Climate Change’s (IPCC) Sixth Assessment Report (AR6) from Working Group II highlights a significant increase in global river and lake temperatures since the 1970s, with increments of 1 °C and 0.45 °C per decade, respectively [8]. This trend is likely to alter sea–land thermal disparities, consequently reshaping large-scale atmospheric circulation patterns and significantly modifying global terrestrial precipitation distributions and patterns [9,10]. Elevated temperatures directly influence climatic extremes by enhancing the atmosphere’s water-holding capacity, which intensifies hydrological cycle processes and increases the frequency and severity of extreme precipitation events [11,12,13].

Substantial evidence suggests that climate change has significantly altered global and regional rainfall patterns [14,15,16]. Some studies have indicated that approximately 11% of global land precipitation has exhibited a “drier gets drier, wetter gets wetter” pattern since the mid-20th century [17,18,19]. Donat et al. utilized observational data and model simulations to conclude that global arid regions have experienced a marked increase in precipitation (1–2%/decade) over the past 60 years, which correlates with rising temperatures; however, there were non-significant precipitation changes in humid regions [20,21]. Studies of global precipitation systems from 2001 to 2020 have revealed diverse characteristics in the internal intensity distribution of precipitation across various spatial scales. The intensity of core areas in small-to-medium precipitation systems has notably declined, with tropical regions experiencing a decrease in core area intensity, while an increase in precipitation intensity was observed in subtropical regions [22,23,24]. Global precipitation has exhibited an expansion and intensification of precipitation centers [25,26,27]. Most regions globally are experiencing increasing disparities in annual precipitation and shifts in seasonal trends as a result of global warming [28,29,30].

In China, researchers have utilized linear trend analysis, 5-year moving averages, cumulative anomaly methods, and other techniques to assess annual, seasonal, and monthly precipitation [31,32]. These studies provide comprehensive insights into the spatiotemporal distribution and evolutionary trends of precipitation within China or specific regions [33,34]. Xu et al. (2020) explored the spatiotemporal distribution of precipitation in China from 1956 to 2018, identifying a non-significant increasing trend with no substantial breakpoints and a primary cycle of 20 years [35]. Zhang et al. (2019) investigated the asymmetric warming of surface soil temperature across China from 1962 to 2011, showing that “the soil diurnal temperature range (SDTR) decreased at most stations (average rate of −0.025 °C/year), with the most profound decrease in winter (−0.08 °C/year)” [36]. Regarding summer precipitation, no significant trend was detected across China from 1957 to 2008, with a decreasing trend observed in the north and an increasing trend in the south. The precipitation in the northwest region of China has shown a cyclical pattern and an overall upward trend. Since the 1980s, precipitation in the northwest region has increased at approximately 0.59 mm/a [37,38], signaling a gradual transition to a warmer and more humid climate condition [39,40]. Tang Yun’s research revealed a downward trend in the annual average precipitation in the northeastern region, decreasing by 0.82 mm/a. This decline is primarily attributed to a reduction in summer precipitation by 0.81 mm/a. The regional variability in precipitation trends is significant, influenced by East Asian monsoons and topographical factors. Smaller decreases in precipitation are observed in the northern region, while more pronounced decreases are evident in the southern region [41,42].

Vegetation, an integral part of terrestrial ecosystems, plays a significant role in climate regulation, soil conservation, and ecosystem stability; however, it is also vulnerable to the impacts of climate change [43,44,45]. The influence of precipitation on vegetation changes is particularly notable, and understanding its effects on vegetation cover is imperative for ensuring ecosystem stability and promoting sustainable regional development [46,47,48]. Research on global water availability and variability has demonstrated that vegetation resilience is more pronounced in regions with higher water availability; however, resilience diminishes in areas characterized by unstable precipitation variability [49,50,51]. Studies utilizing the Normalized Difference Vegetation Index (NDVI) have revealed that increased precipitation positively impacts grassland vegetation growth outside the Arctic [52,53,54,55]. Research spanning from 1982 to 2019 in China’s southwest region has highlighted distinct spatial variations in the influence of precipitation on vegetation cover [56,57,58]. These studies have identified significant positive correlations between growing season precipitation and vegetation cover [59,60,61,62,63].

Previous research on the impacts of precipitation on global and regional climate and vegetation has predominantly focused on monthly and annual precipitation data. Continuous precipitation is defined as precipitation characterized by a prolonged duration and little variation in intensity. It typically originates from nimbostratus or thick, low-altitude altostratus clouds. Wang and Li provided a methodological foundation for the statistical analysis of precipitation processes in weather forecasting [64]. However, the condition of continuous precipitation is crucial for elucidating precipitation evolution patterns and improving forecast accuracy. Especially in arid regions, it is scientifically vital for the effective prevention and mitigation of weather-related hazards. Despite its importance, the characteristics of continuous precipitation remain uncertain, both at global and regional levels.

As a consequence, this study attempts to establish a set of continuous precipitation indices, including $CP_{d}$ (continuous precipitation days); $ACP_{t}$ (annual continuous precipitation times); $CP_{a}$ (continuous precipitation amounts); and FCP (frequency in different ranges of $ACP_{a}$). We utilized data from 1991 to 2020, incorporating observational precipitation data and the Normalized Difference Vegetation Index (NDVI), to explore the spatiotemporal characteristics of continuous precipitation and its relationship with vegetation cover in China.

The objective is to enhance our understanding and predictive capabilities regarding the water adjustment efficacy of continuous precipitation on vegetation, instead of focusing solely on accumulated precipitation. By illuminating the intricate relationship between vegetation and climate change at the national level, the findings of our study can offer valuable new insights for ecological management and conservation efforts.

## 2. Materials and Methods

### 2.1. Study Region

**Figure 1:**
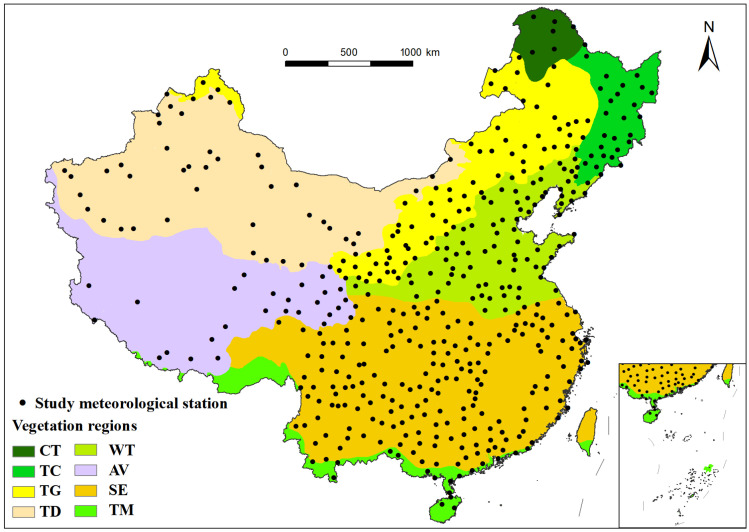
Distribution of meteorological stations used in this study. Note: This map was produced using the 1:1,000,000 National Fundamental Geographic Database (National Geomatics Center of China; Map Review No.: GS (2016) 2556) with unmodified base map boundaries. The CN-border dataset was processed by the GMT Chinese Community based on this database.

China is located in Eastern Asia, bordering the Western Pacific Ocean (73°33′ E to 135°05′ E, 3°51′ N to 53°33′ N), and is characterized by a complex and diverse range of climatic types. Utilizing the vegetation zoning data provided by the Data Center for Resources and Environmental Sciences, Chinese Academy of Sciences, the research area was divided into eight distinct vegetation regions (Figure 1). These regions include cold temperate coniferous forest (CT), temperate coniferous and deciduous broad-leaved mixed forest (TC), temperate grassland (TG), temperate desert (TD), warm temperate deciduous broad-leaved forest (WT), alpine vegetation of Qinghai–Tibet Plateau (AV), subtropical evergreen broad-leaved forest (SE), and tropical monsoon forest and rainforest (TM) [65,66,67].

### 2.2. Data

Precipitation data for this study was obtained from the Chinese Surface Climate Data Daily Dataset (V3.0), available through the China Meteorological Data Network (http://data.cma.cn (accessed on 7 August 2022)), for the period from 1991 to 2020. In this study, precipitation measurements (in mm) were taken from observation stations with bare soil. Precipitation was recorded on an accumulation basis from across 24 h to calculate daily precipitation. The Normalized Difference Vegetation Index (NDVI) data from 1991 to 2020 (March to the following November) were provided by Geographic remote sensing ecological network platform (www.gisrs.cn (accessed on 24 October 2024)), which were derived from MODIS (Moderate-Resolution Imaging Spectroradiometer) products with a spatial resolution of 4000 m. The base map of China’s national boundaries used in this study was derived from the 1:1,000,000 National Fundamental Geographic Database (2021 edition) provided by the National Geomatics Center of China, and was processed into the CN-border dataset by the GMT Chinese Community.

### 2.3. Methods

#### 2.3.1. Precipitation Data Processing

The original dataset of Chinese precipitation encompassed records from 759 national standard meteorological stations. However, due to the presence of incomplete data regarding dates and specific measurements at certain stations, we adopted a rigorous criterion prioritizing the completeness of annual records at each station. This criterion reduced the number of stations to 467, which were subsequently categorized into eight parts based on the above vegetation regional division (Figure 1). Utilizing the ArcGIS Pro version 3.5 (Esri, Redlands, CA, USA), an overlay analysis was conducted between the selected meteorological station layers and the vegetation region layers. This resulted in a spatial distribution of meteorological stations based on vegetation regions.

In this study, consecutive precipitation events—where a rainfall event is defined as a day with precipitation exceeding 0.01 mm—were identified, and we defined CP as continuous precipitation (an event of precipitation every day during a period of time). Continuous precipitation days (days in one event of continuous precipitation) are denoted as $CP_{d}$, while the total precipitation amount over a continuous K period, referred to as the continuous precipitation amounts, is noted as $CP_{a}$. Consequently, the count of K is the annual continuous precipitation times $ACP_{t}$ for a given station in a given year. $MACP_{t}$, the average value of K, represents the multiyear mean of annual continuous precipitation times. $MMCP_{t}$, the maximum value of K, is the multiyear maximum of annual continuous precipitation times. The average value of $CP_{a}$, $MACP_{a}$, is the multiyear mean of continuous precipitation amounts. The maximum value of $CP_{a}$, $MMCP_{a}$, is the multiyear mean of annual maximum of continuous precipitation amounts.

The SAS version 9.4 (SAS Institute Inc., Cary, NC, USA) was used to calculate the continuous precipitation days $CP_{d}$ and the continuous precipitation amounts $CP_{a}$ for 467 stations over 30 years, as well as the maximum value of K, $MMCP_{t}$, and the maximum value of $CP_{a}$, $MMCP_{a}$. For each station, the 30-year average values were determined. The regional average value was calculated based on the data of stations included in the corresponding vegetation region.

For precipitation data of each station in each year, $CP_{a}$ is categorized into six intervals as follows:

(1) $$b_{i}=\left\{\begin{array}{l} \mathrm{int}(CP_{a},5)\;0<CP_{a}<5\\ \mathrm{int}(CP_{a},5)\;5\leq CP_{a}<10\\ \mathrm{int}(CP_{a},10)+1\;10\leq CP_{a}<20\\ \mathrm{int}(CP_{a},10)+1\;20\leq CP_{a}<30\\ \mathrm{int}(CP_{a},10)+1\;30\leq CP_{a}<40\\ \mathrm{int}(CP_{a},10)+1\;40\leq CP_{a}<50\\ \mathrm{int}(CP_{a},50)+5\;50\leq CP_{a}<100\\ \mathrm{int}(CP_{a},50)+5\;100\leq CP_{a}<150\\ b_{i-1}+1\;CP_{a}\geq 150 \end{array}\right.$$

where b is the interval of cumulative precipitation, with larger b values indicating more significant precipitation amounts. Each continuous precipitation interval corresponds to a $CP_{a}$; thus, every time the same continuous precipitation interval was counted, they were converted into percentages to ascertain the prevalence of each precipitation interval. For each meteorological station, a 30-year average value is computed. For each vegetation region, the regional average value is determined based on the averages of the stations included within that region. This approach provides a methodical means to examine precipitation patterns in relation to various vegetation types over an extensive temporal scope.

#### 2.3.2. Processing of NDVI Dataset

In this paper, the Normalized Difference Vegetation Index (NDVI) data spanning from 1991 to 2020 was applied to explore the spatiotemporal change in vegetation cover. Based on the annual NDVI dataset from 1991 to 2020, the change trend of NDVI in 30 years was calculated pixel-by-pixel in the unary linear regression method. The calculation formula is as follows:

$$\theta_{slope}=\frac{\left(n\times \sum_{i=1}^{n}i\times X_{i})-(\sum_{i=1}^{n}i\sum_{i=1}^{n}X_{i}\right)}{n\times \sum_{i=1}^{n}i^{2}-{\left(\sum_{i=1}^{n}i\right)}^{2}}$$

In the formula, $\theta_{slope}$ indicates the pixel-by-pixel change trend of NDVI, where the pixel NDVI tended to increase potential when $\theta_{slope}>0$, while $\theta_{slope}<0$ indicates the completely opposite state, which means the pixel NDVI shows a downward trend; n is the number of years (30 years); i is the serial number of a year in 30 years; and $X_{i}$ is the NDVI value corresponding to year i.

In order to have a discussion on the spatiotemporal variation in vegetation cover in combination with the changing trend of NDVI, the annual NDVI dataset from 1991 to 2020 was divided into three 10-year periods for contrastive analysis with the Mean Value Composite—there would be average NDVI from 1991 to 2000, average NDVI from 2001 to 2010, and average NDVI from 2011 to 2020 in the fruits.

#### 2.3.3. Processing of Correlation Analysis

The multiple precipitation data were imported into ArcGIS software, which includes $MP_{a}$, $MCP_{d}$, $MMCP_{d}$, $MACP_{a}$, $MMCP_{a}$, and $MACP_{t}$. These datasets were harmonized within the unified projection coordinate system with NDVI. To perform NDVI and multiple precipitation data correlation analysis, spatial interpolation processing was employed to process a variety of precipitation data to obtain raster data sets with the same spatial resolution as NDVI data. A detailed pixel-scale correlation analysis was executed between NDVI and various precipitation data. The correlation coefficients for each grid point were computed using a specific formula and were visually represented on a map.

(2) $$R_{xy}=\frac{\sum_{i=1}^{n}[(x_{i}-\bar{x})(y_{i}-\bar{y})]}{\sqrt{\sum_{i}^{n}(x_{i}-\bar{x}}{)}^{2}\cdot \sqrt{\sum_{i=1}^{n}{\left(y_{i}-\bar{y}\right)}^{2}}}$$

This formula incorporates elements denoting the correlation coefficient $R_{xy}$ between variables x and y; the NDVI value $x_{i}$ for year i; the measurement $y_{i}$ for variables precipitation data in millimeters for year i; and the mean values $\bar{x}$ and $\bar{y}$ for NDVI and variables over a period of n years [68].

## 3. Results

### 3.1. Spatiotemporal Characteristics of Annual Precipitation Amounts in China from 1991 to 2020

**Figure 2:**
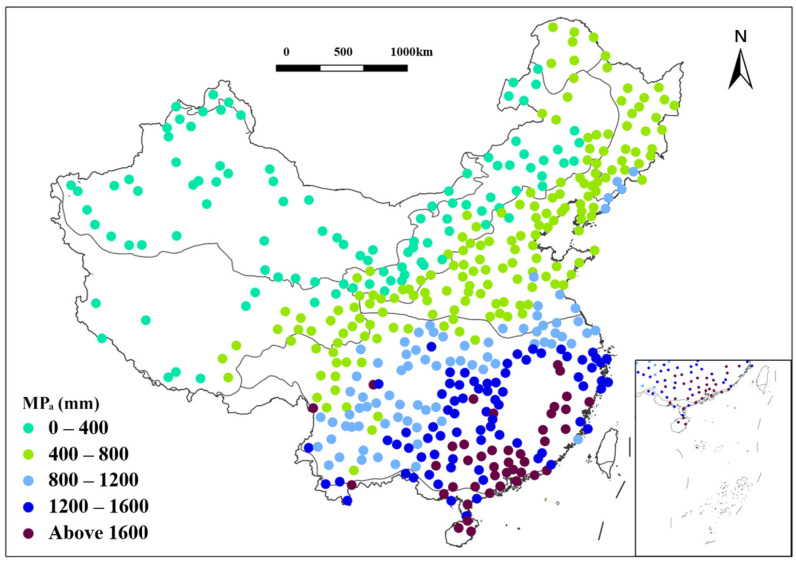
Distribution of multiyear mean of annual precipitation amount (MP_a_) of each vegetation region in China from 1991 to 2020. Circles represent 467 meteorological stations, with each color indicating a different range of MP_a_.

The national average of the multiyear means of annual precipitation amounts ($MP_{a}$) was 823.5 mm, showing a general trend of gradual decrease from the southeastern coastal regions toward the northwestern inland areas. The highest regional average of $MP_{a}$ was recorded in the TM region, reaching 1778.4 mm; conversely, the lowest regional average of $MP_{a}$, at 123.2 mm, was observed in the TD region (Figure 2 and Table 1).

In CT region, the $MP_{a}$ exhibited a uniform distribution, with the highest $MP_{a}$ being 552.6 mm and the lowest at 439.3 mm. Within this region, specifically east of the Greater Khingan Range, the $MP_{a}$ ranged between 440 and 550 mm. The greatest $MP_{a}$ values were recorded in the northern and southeastern parts of the TC region, with precipitation in the western area significantly less than in the eastern area. Of the 27 stations in this region, those located east of the Songhua River basin had $MP_{a}$ exceeding 600 mm, while those located to the north usually ranged between 500 mm and 600 mm. While the TG region was generally characterized by lower precipitation, its northern part, adjacent to the forests, experienced comparatively higher levels. The 30-year $MP_{a}$ data for the expansive TD region ranged from 14.4 mm to under 200 mm, with a median below 100 mm, indicating overall stability. In the WT region, the eastern area experienced an $MP_{a}$ ranging between 600 and 900 mm, while the areas north of the Hexi Corridor received between 300 and 450 mm. The AV region generally experienced $MP_{a}$ between 300 and 700 mm, with the lowest being only 70.5 mm.

Over the past 30 years, the regional average $MP_{a}$ was 450 mm, with data concentrated in the eastern central plains of the AV region, where the maximum reached 763.1 mm. The SE region exhibited a wide range of values, from 470.2 mm to 2445.3 mm, the highest in the study area. In the Wujiang River basin and surrounding areas, $MP_{a}$ frequently exceeded 1000 mm, compared to over 1200 mm in southeastern coastal zones, while the western part recorded only 400–800 mm. Among 467 meteorological stations, the maximum observed $MP_{a}$ was 2445.3 mm. The TM region recorded an average of 1778.4 mm and a peak of 2424.6 mm, with all values exceeding 1300 mm, consistent with its tropical coastal climate.

### 3.2. New Precipitation Index System

#### 3.2.1. Spatiotemporal Characteristics of Continuous Precipitation Days in China from 1991 to 2020

**Figure 3:**
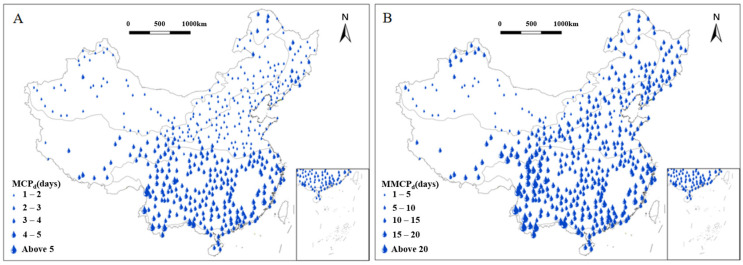
Distribution of multiyear mean of continuous precipitation days (MCP_d_) of each vegetation region in China from 1991 to 2020; water drops represent 467 meteorological stations, with each size indicating a different range of MCP_d_ (**A**). Distribution of multiyear mean of annual maximum of continuous precipitation days (MMCP_d_) of each vegetation region in China from 1991 to 2020; water drops represent 467 meteorological stations, with each size indicating a different range of MMCP_d_ (**B**).

The national average of the multiyear means of continuous precipitation days ($MCP_{d}$) was approximately 2.3 days (Figure 3A and Table 1). The TM region had the highest average $MCP_{d}$ at 3.2 days, whereas the TD region had the lowest, averaging at 1.5 days.

In the CT region, the values of $MCP_{d}$ were generally short, with a regional average of 2.1 days. This was particularly evident in the eastern and southern parts of the region, where $MCP_{d}$ was approximately just 1 day. The $MCP_{d}$ in the TC region ranged from 1 to 3 days, with the maximum values occurring south of the Changbai Mountains. For the $MCP_{d}$ in the TG region, the highest values were found in the northwestern regions adjacent to the Greater Xing’an Mountain range. The spatial distribution of $MCP_{d}$ in the TD region was greatly influenced by proximity to marine and land areas, with most regions averaging less than 2 days while the $MCP_{d}$ near the Erqis River reached up to 2 days. In the WT region, the southern parts exhibited $MCP_{d}$ between 2 and 3 days, with most other areas ranging from 1 to 2 days. Similarly, in the AV region, the eastern part had the largest proportion of $MCP_{d}$ within 2–3 days, with a minority between 1 and 2 days; the southern regions also averaged 2–3 days. In the SE region, the Sichuan Basin recorded $MCP_{d}$ of more than 5 days, while the $MCP_{d}$ in the western regions and southeastern coastal areas were mainly concentrated between 3 and 4 days, with the central areas mostly ranging from 2 to 3 days. In the TM region, the maximum observed $MCP_{d}$ was 4 days. The Southern Yunnan–Guizhou Plateau exceeded 4 days, and the rest of the region generally ranged between 3 and 4 days.

The national average of the multiyear means of annual maximum of continuous precipitation days ($MMCP_{d}$) stands at 9 days (Figure 3B and Table 1). Of the eight vegetation regions, the highest regional average was recorded in the TM region, reaching approximately 15 days. The TD region had the lowest average value of $MMCP_{d}$ at only 4.4 days.

In the CT region, $MMCP_{d}$ exhibited a balanced distribution, predominantly ranging from 6 to 10 days, with a regional maximum and average of 10 and 8 days, respectively, indicating minimal intra-regional variability. The TC region exhibited a similar range and average, also reflecting minor internal differences. For the TG region, despite its large north–south extent, $MMCP_{d}$ was generally low, though some southern and northern stations reached 6–10 days, forming a pattern of lower values in the center. In the TD region, durations were mainly concentrated between 2 and 6 days, with a regional average of just 4.4 days—the lowest among all eight zones—though some northwestern and southeastern stations reached 6–10 days. The WT region displayed a significant north–south gradient, as most southern areas recorded 6–10 days, while northern areas were mostly 2–6 days, showing a general decrease from south to north. The AV region exhibited clear east–west differences, with the east mainly experiencing 10–14 days and the west only 2–6 days. In the SE region, $MMCP_{d}$ generally ranged from 6 to 10 days but fluctuated westward to east, as the west saw extreme values exceeding 18 days, the center had fewer occurrences, the southeast notably increased to 10–14 days, while the northeast remained at 6–10 days. The TM region, narrowly distributed along the southern coast, had the highest $MMCP_{d}$ among all regions, averaging 15 days and peaking at 27 days, with a substantial east–west increase—western areas mostly exceeded 18 days, compared to 6–14 days in the east.

#### 3.2.2. Spatiotemporal Characteristics of Continuous Precipitation Amounts in China from 1991 to 2020

**Figure 4:**
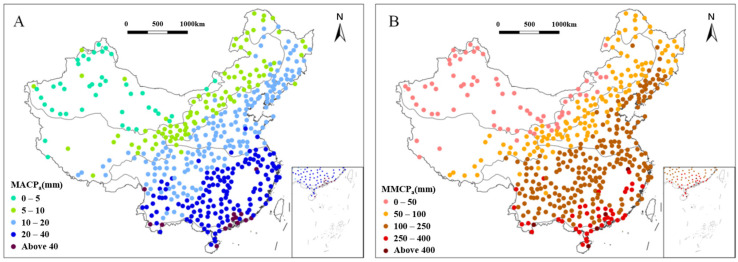
Distribution of multiyear mean of continuous precipitation amount (MACP_a_) of each vegetation region in China from 1991 to 2020; circles represent 467 meteorological stations, with each color indicating a different range of MACP_a_ (**A**). Distribution of multiyear mean of annual maximum of continuous precipitation amount (MMCP_a_) of each vegetation region in China from 1991 to 2020; circles represent 467 meteorological stations, with each color indicating a different range of MMCP_a_ (**B**).

The average of the multiyear means of continuous precipitation amounts ($MACP_{a}$) in China was 16.7 mm (Figure 4A and Table 1). Among the eight vegetation regions, the TM region exhibited the largest average value of $MACP_{a}$ at 37.1 mm, with the TD region holding the lowest at just 3.8 mm.

The CT region, located closer to the semi-humid regions, particularly to the southeast of the Greater Xing’an Mountains, reached $MACP_{a}$ values of 8 to 11 mm, while the northern and southwestern parts generally ranged between 6 and 8 mm. Among the 27 stations in the TC region, those located east of the Songhua River basin mostly had $MACP_{a}$ values exceeding 10 mm, while those located to the north mostly ranged within 8 to 11 mm. The southwestern and central areas of the TG region experienced relatively less precipitation, with $MACP_{a}$ typically below 10 mm. The northern areas, being closer to forests, had more precipitation, ranging between 8 and 15 mm. The TD region spanned a broad east–west area, but most $MACP_{a}$ values were less than 6 mm. Half of the areas had $MACP_{a}$ below 3 mm, with the lowest $MACP_{a}$ reaching 1.3 mm—the lowest within the eight regions. The eastern area of the WT region had $MACP_{a}$ values mostly between 8 and 22 mm, while the western areas, including the Inner Mongolia Plateau and north of the Hexi Corridor, ranged from 8 to 12 mm. $MACP_{a}$ of the AV region ranged from 1 to 15 mm, with the lowest recorded at 3.9 mm. The current dataset for the AV region is concentrated in the southern and eastern areas, close to the Central Plains region, where the highest $MACP_{a}$ recorded is 15.1 mm. Accounting for the largest proportion of China, the SE region had a wide range of variations in $MACP_{a}$, ranging from 9 to 47 mm. The southeastern coastal areas of the SE region mostly had $MACP_{a}$ values above 32 mm, while the western regions, influenced by their proximity to the sea, ranged from approximately 8 to 22 mm. The TM region had an average $MACP_{a}$ of 37.1 mm, with all observations located above 27 mm, peaking at 51.3 mm, which was the highest value recorded among the eight regions.

The national average of the multiyear means of annual maximum of continuous precipitation amounts ($MMCP_{a}$) was 132.8 mm (Figure 4B and Table 1). In all eight regions across China, the $MMCP_{a}$ generally exhibited a decreasing trend from the southeast toward the northwest. The highest regional average of $MMCP_{a}$ was observed in the TM region at 346 mm, while the lowest was found in the TD region, reaching only 24 mm.

The CT region, primarily located in the northern part of the Greater and Lesser Xing’an Mountains, consistently exhibited values of $MMCP_{a}$ between 60 and 80 mm. The TC region exhibited $MMCP_{a}$ values mainly concentrated between 70 to120 mm, demonstrating a gradual decreasing trend from south to north. The $MMCP_{a}$ in the TD region was generally low, ranging from 5 to 60 mm, consistent with the low precipitation characteristic of temperate continental climates. The regional trends in the WT region generally showed a decrease from the coast to the inland, with the southern area exhibiting higher $MMCP_{a}$ values (120 to 180 mm) than that of the north (50 to 100 mm); however, higher values of 150 to 200 mm were observed in the northeastern coastal areas. The AV region displayed significant differences compared to adjacent vegetation regions at the same latitude. $MMCP_{a}$ ranged predominantly between 50 and 100 mm, with lower values in the western regions. The SE region generally had higher $MMCP_{a}$ values. In the eastern, southern, and western areas, values greater than 240 mm were observed, while the central area mainly ranged between 120 and 180 mm, with occasional instances exceeding 180 mm. Values below 120 mm were mostly distributed in the western area, indicating significant regional disparities and a general trend of decreasing from southeast to northwest. The TM region was primarily located in the southern coastal areas, with a narrow latitudinal range and regional differences. $MMCP_{a}$ values were all greater than 240 mm, indicative of abundant precipitation.

#### 3.2.3. Spatiotemporal Characteristics of Continuous Precipitation Times in China from 1991 to 2020

**Figure 5:**
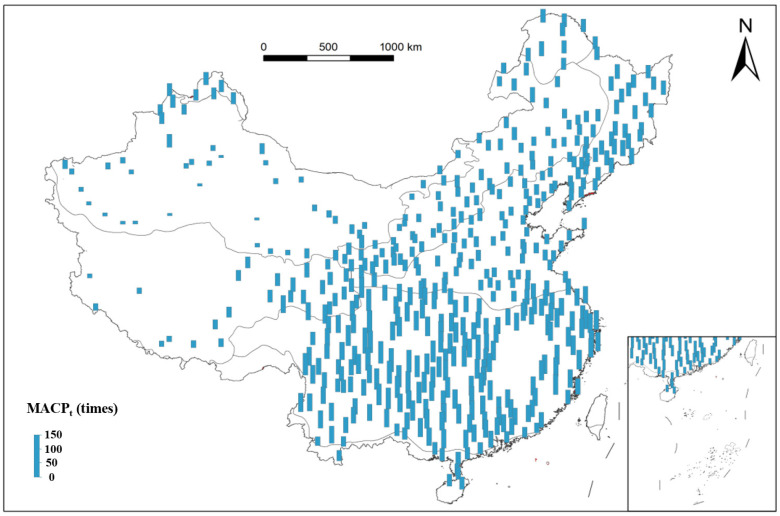
Distribution of multiyear mean of annual continuous precipitation times (MACP_t_) of each vegetation region in China from 1991 to 2020. Each bar corresponds to one of the 467 meteorological stations, with each length indicating a different range of MACP_t_.

In China, the average value of the multiyear means of annual continuous precipitation times ($MACP_{t}$) was 57.7 (Figure 5 and Table 1). Throughout the eight vegetation regions, the trend of the $MACP_{t}$ showed a gradual decrease from south to north. The highest regional average was recorded in the SE region at 77.9 times. In contrast, the TD region exhibited the lowest regional average, amounting to only 29.8 times.

In the CT region, $MACP_{t}$ variability was relatively low, averaging 56 times—slightly below the national average—with a maximum recorded value of 62 times. The TC region surpasses the national average with a mean $MACP_{t}$ of 59 times. By contrast, the TG region had a lower average of 43 times, though northern and southeastern areas experienced higher values, ranging between 40 and 60 times. The TD region showed notably low averages, at only 29.8 times, with high internal variability as values spanned from 10 to 56 times. Higher counts occurred in northwestern mountainous areas, generally decreasing eastward. The WT region had an average of 44 times, below the national level, with limited variability. Counts were slightly elevated in the northeast and southeast, and declined toward the west. The AV region displayed significant east–west differences, as eastern areas reached up to 62 times, while western areas were lower, reaching as low as 18 times, illustrating a distinct east-high, west-low pattern. The SE region exhibited the highest values nationally, with a regional average of 78 times and a peak of 109 times. High values clustered in the west and gradually decreased southward. The TM region averaged 49 times, with slightly higher values in the east and a general westward decline.

**Figure 6:**
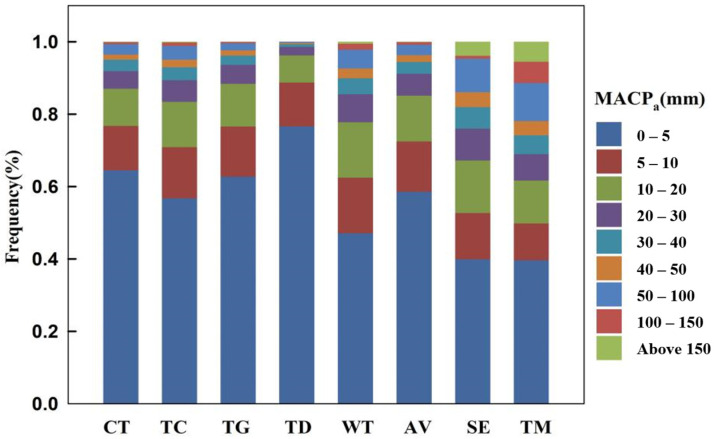
Frequency of MACP_a_ level under eight vegetation regions of China from 1991 to 2020. Nine colors within each bar denote the frequency distribution of the MACP_a_ in nine precipitation ranges; for example, in the CT region, the occurrences of MACP_a_ in 0–5 mm accounts for 64.7% of the total times of MACP_a_.

Across the eight vegetation regions, the frequency of continuous precipitation events decreased with the increase of $MACP_{a}$ (Figure 6). The occurrence of MFCP (frequency in different ranges of $MACP_{a}$) in the 0–5 mm range was significantly greater than that in other ranges, with values exceeding 50% in the CT, TC, TG, TD, and AV regions, reaching as high as 76.7% in the TD region. Conversely, they were slightly less than half in the WT, SE, and TM regions, and reached as low as 40%. Occurrences of $MACP_{a}$ above 150 mm were relatively rare across the vegetation regions and thus constituted a minimal MFCP. There were no occurrences of $MACP_{a}$ exceeding 150 mm in the TD region; conversely, there were frequent occurrences in the same range of $MACP_{a}$ in the SE and TM regions, representing a comparatively larger frequency.

In the CT region, occurrences of $MACP_{a}$ less than 20 mm accounted for over 80% of the region, while MFCP in the 0–5 mm range of $MACP_{a}$ were the most frequent, accounting for 64.5% of the region. Events exceeding 150 mm were rare, accounting for only 0.1% of occurrences. In the TC region, the MFCP within the 0–5 mm range of $MACP_{a}$ accounted for 56.8% of precipitation. The remaining precipitation was primarily concentrated between 5 and 20 mm, making up 26.6% of the total, while instances exceeding 20 mm represented 16.6%. In the TG region, $MACP_{a}$ below 20 mm comprised a significant majority, constituting 88% of the region. In this region, the frequency of $MACP_{a}$ levels exceeding 20 mm was markedly lower than those in most of China. The TD region had the highest frequency (76.7%) of $MACP_{a}$ at the 0–5 mm level across China. The MFCP of $MACP_{a}$ values below 20 mm also constituted the largest percentage, with no events exceeding 150 mm recorded in the TD region. In contrast, the WT region had less than 50% of events in the 0–5 mm range, over 30% in 5–20 mm, and a significantly higher frequency of events above 20 mm than the other regions. The AV region recorded 58.6% with the TC region in the 0–5 mm range, with similar MFCP values observed across three ranges: 5–10 mm; 10–20 mm; and above 20 mm. The SE region was one of three vegetation regions where the frequency of $MACP_{a}$ in the 0–5 mm range was 39.9%. This resulted in the proportion of $MACP_{a}$ values exceeding 5 mm in this region being higher than in other regions, where the 50–100 mm range was notable (9.2%) and events exceeding 150 mm accounted for 3.9%, a relatively high proportion nationally. In the TM region, the proportion of 0–5 mm $MACP_{a}$ events was the lowest among the eight regions, at just 39.7%, with a more uniform distribution across the ranges, a higher share of events over 50 mm, and the highest proportion of events exceeding 150 mm.

### 3.3. The Mean NDVI Changes in China from 1991 to 2020

**Figure 7:**
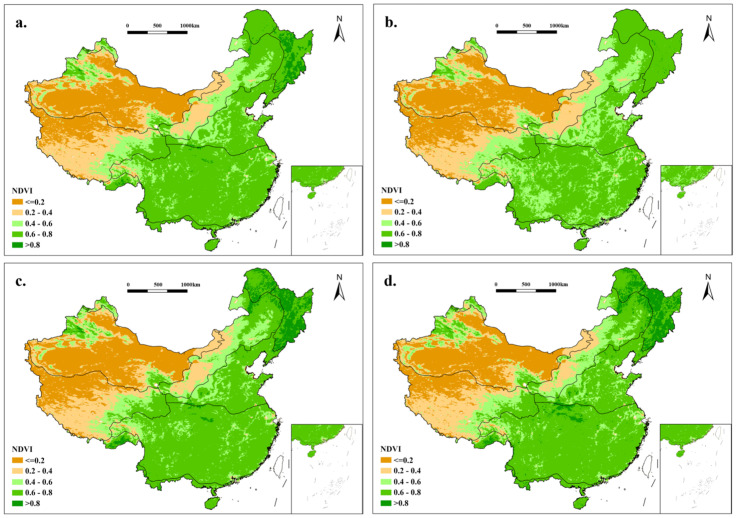
(**a**) The average NDVI of different vegetation zones in China from 1991 to 2020. (**b**) The average NDVI of different vegetation zones in China from 1991 to 2000. (**c**) The average NDVI of different vegetation zones in China from 2001 to 2010. (**d**) The average NDVI of different vegetation zones in China from 2011 to 2020.

The mean NDVI of the CT region was approximately 0.56 with a good trend in plant growth, as there were high vegetation cover and abundant forest resources (Figure 7). The NDVI value of the TC region recorded in the area reached 0.79, and the vegetation in the northeast and southwest of the area grew significantly, with plants growing better on the eastern side of the Changbai Mountains than on the western side. The mean NDVI of the TG region was 0.54 over 30 years. The northern and eastern regions exhibited higher NDVI values than the southern and western areas. The NDVI improved markedly, reflecting the enhanced grassland health and gradual restoration of ecological functions. The TD region had the lowest NDVI average of 0.24, with limited change in numbers and a correlation coefficient of 0.02, which is far below the national average. The correlation coefficient was up to 0.70 in the WT region, with huge improvements in the North China Plain and parts of the Loess Plateau. The region’s plant coverage was concentrated in the Central Belt, and the majority of NDVI values were greater than 0.4. The correlation coefficient was 0.2 in the AV region. The area is characterized by a very heterogeneous distribution of plant covering, with plant density decreasing from southwest to northeast displaying this pronounced spatial heterogeneity. The SE region, as the largest climate zone, had a mean NDVI of 0.66 and a growth rate of 0.003 per decade. Lower NDVI values occurred in the Yangtze River Delta and high-altitude western areas (Qinghai–Tibet Plateau), while higher values were observed in northern regions influenced by the Qinling Mountains. Its correlation coefficient was as high as 0.86 and ranked highest among all the zones. Conversely, the smallest climate zone, the TD region, had a 0.66 high mean NDVI and stable trends, with a correlation coefficient of 0.86 as the highest. Vegetation density improved substantially over 30 years, particularly in the Hengduan Mountains.

**Figure 8:**
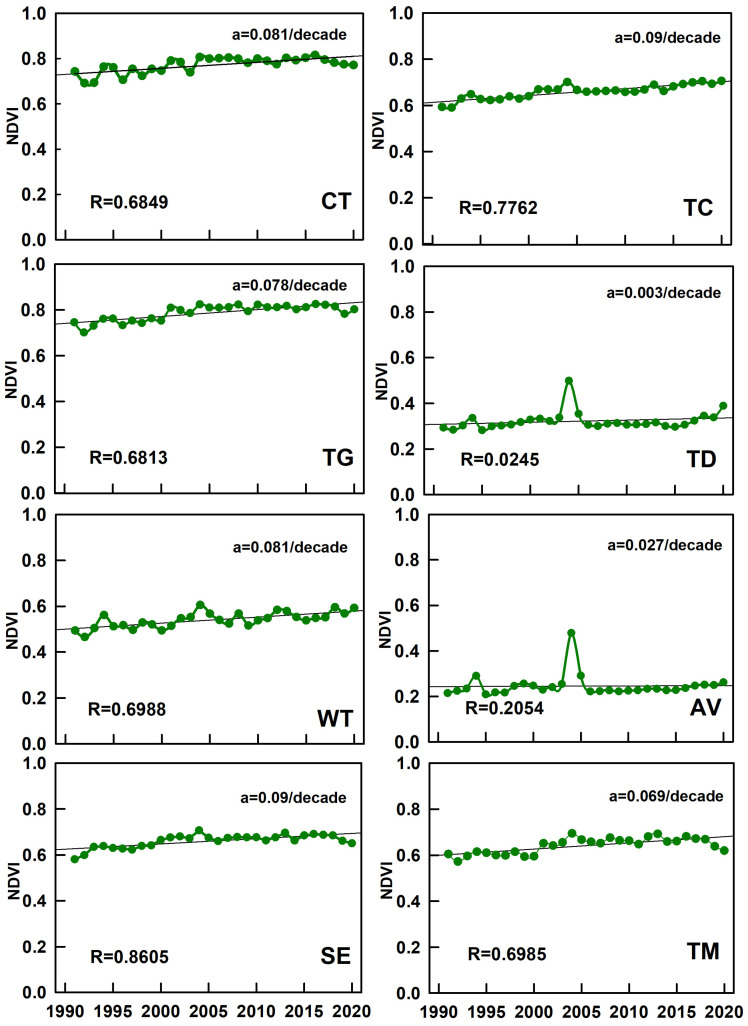
The temporal distribution of NDVI in various vegetation areas of China from 1991 to 2020. Each green point corresponds to the mean NDVI of the given vegetation zone in that specific year, the line represents the fitted trend of NDVI change. An overall upward trend indicates an improvement in vegetation cover, whereas a downward trend indicates degradation.

**Figure 9:**
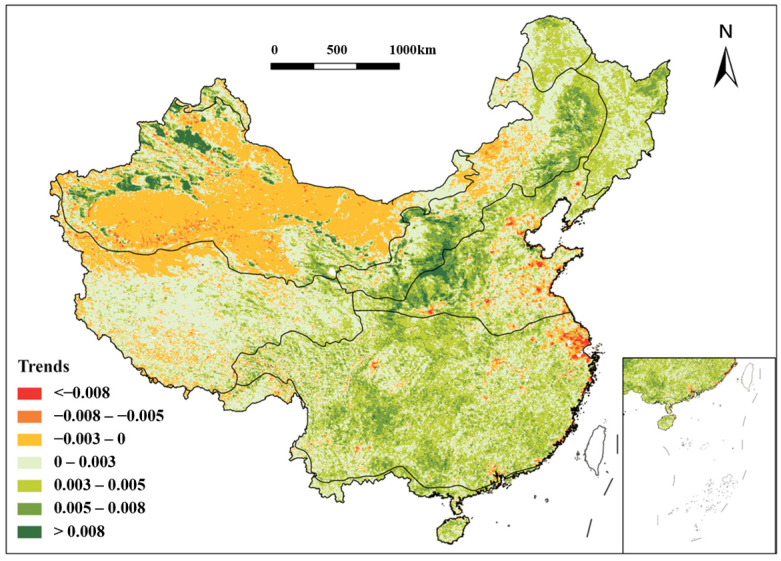
The spatial distribution of NDVI variation trends in various vegetation areas of China from 1991 to 2020.

In the CT region, there was a 7% increase in NDVI values in 2010 over a decade ago, and NDVI values were greater than 0.8 in most areas in 2010 (Figure 8). The vast majority of NDVI values in the TC region were greater than 0.8, and the vegetation cover was relatively stable. The vegetative coverage in the TG region has increased, with an increase of about 8.9% in NDVI values in 2010 compared to 2000, and an increase of about 10.1% in 2020 compared to 2010. The change in vegetation cover is extremely pronounced in the central and southern regions, where the areas with lower NDVI values are significantly reduced. In the TD region, vegetation coverage was more stable over the 30-year period, and the concentration of low vegetation cover within the region has improved (Figure 9). In 2010, there was an increase of about 11.4% from the NDVI values of a decade earlier in the WT region, but the values have rebounded after a decade. In alpine vegetation region of Qinghai–Tibet Plateau, there was an approximate 27.2% increase in NDVI values from 2020 compared to 2010, with a significant increase in the size of the area of high vegetation cover, particularly in the northwestern portion of the district. Since 1991, the growth rates of NDVI values per decade have been 7.8%, 2.9%, and 7.2% in the subtropical evergreen broad-leaved forest region. The vegetation cover has continued to improve, and the growth of NDVI values has been significant throughout the region, especially in the central region. NDVI increased by 14.5% from 1991 to 2000 in the TM region compared to the more stable 2010 and 2020 data, and consistently ranked among the highest nationally.

### 3.4. Effect of Continuous Precipitation Times on Vegetation Cover in China from 1991 to 2020

#### 3.4.1. Impact of Multiyear Average Maximum Consecutive Precipitation Amount (MMCP_a_) on Vegetation Coverage

**Figure 10:**
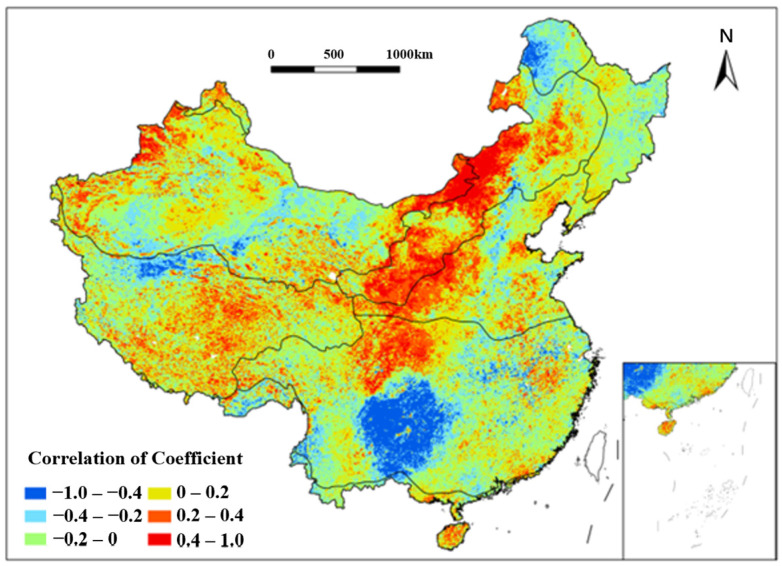
Spatial distribution of correlation coefficient between NDVI and MMCP_a_ in China from 1991 to 2020. Each color indicates a range of correlation coefficient between NDVI and MMCP_a_.

Analysis of the NDVI and multiyear average maximum consecutive precipitation amount ($MMCP_{a}$) revealed extremely strong negative correlations (−1.0 to −0.6) in the SE regions east of the Hengduan Mountains and south of the Qinling Mountains from 1991 to 2020 (Figure 10). These areas, under subtropical monsoon climates, experience excessive rainfall that fosters fungal/bacterial diseases and pests, hindering vegetation growth. Additional negative correlations were observed in the Northern Greater Khingan Mountains (CT), Southern Kunlun Mountains (AV), and the Yangtze River mid-lower reaches during the study period. Conversely, strong positive correlations were found in the Loess Plateau (SE, TM) and TD/TG regions east of the Yin Mountains and west of the Greater Khingan Mountains from 1991 to 2020. $MMCP_{a}$ thus exhibits spatially divergent effects on vegetation.

#### 3.4.2. Impact of Multiyear Average Consecutive Precipitation Events (MACP_t_) on Vegetation Coverage

**Figure 11:**
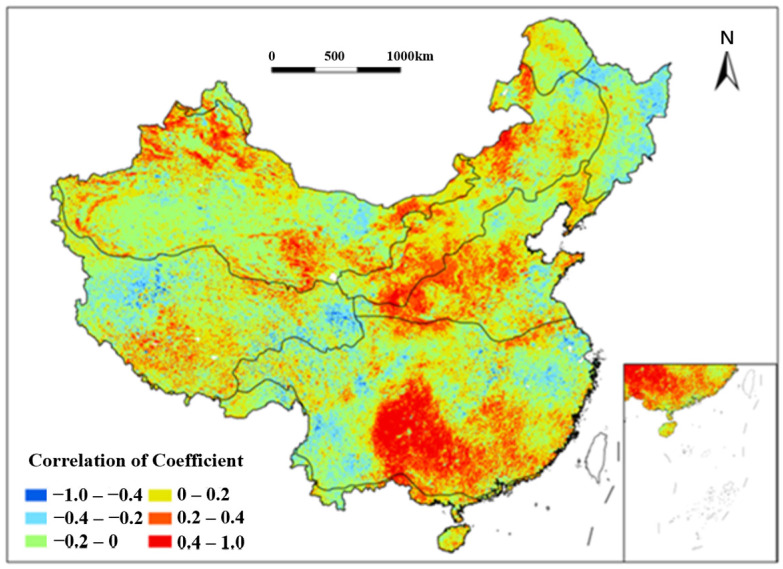
Spatial distribution of correlation coefficient between NDVI and MACP_t_ in China from 1991 to 2020. Each color indicates a range of correlation coefficient between NDVI and MACP_t_.

A correlation analysis between NDVI changes and the multiyear average consecutive precipitation events ($MACP_{t}$) in China was conducted from 1991 to 2020 (Figure 11). The results indicate that NDVI and $MACP_{t}$ exhibited strong positive correlations in the central and southern parts of the SE region over the past three decades, covering extensive and concentrated areas. Notably, parts of the Yunnan–Guizhou Plateau showed correlation coefficients between 0.6 and 1.0 from 1991 to 2020, reflecting extremely strong positive correlations. Smaller pockets of similarly strong positive correlations were sporadically distributed within areas of moderate correlation from 1991 to 2020, including parts of the Qinling Mountains (TC and TG regions), the Northern Greater Khingan Mountains (TC), the Junggar Basin, the Qaidam Basin (TD), and the Northern Tibetan Plateau (AV). Conversely, strong negative correlations (mostly below −0.6) were observed in scattered areas west of the Tibetan Plateau, the Northern Hengduan Mountains (AV), parts of the Lesser Khingan Mountains (TC), and the Lancang River Basin (SE) from 1991 to 2020. Notably, the Eastern Bayan Har Mountains exhibited extremely strong negative correlations (−1.0 to −0.6) during the study period. These findings suggest a significant correlation between NDVI and $MACP_{t}$. The Yunnan–Guizhou Plateau and surrounding areas, characterized by a subtropical monsoon climate with hot, rainy summers and mild, dry winters, experience frequent consecutive precipitation events, providing optimal temperature and humidity for vegetation growth from 1991 to 2020. This aligns with the observed strong positive correlations, indicating that $MACP_{t}$ promotes vegetation growth through sustained water availability.

#### 3.4.3. Impact of Multiyear Average Maximum Consecutive Precipitation Days (MMCP_d_) on Vegetation Coverage

**Figure 12:**
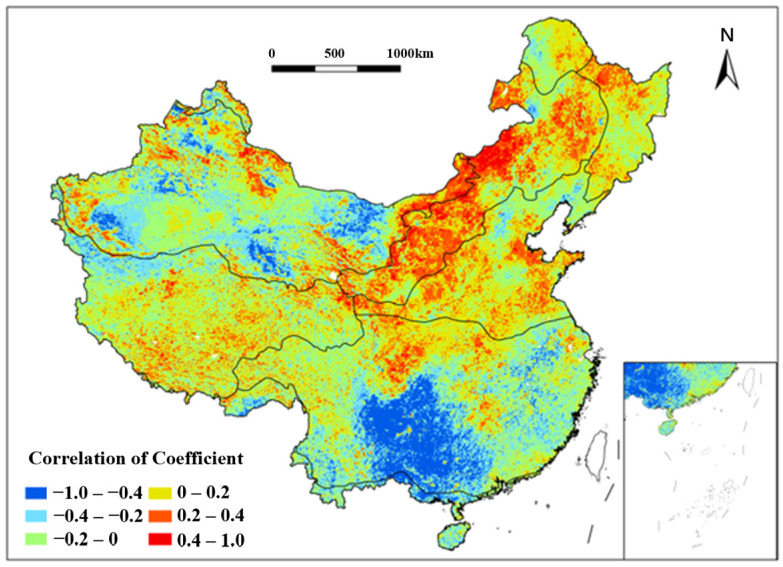
Spatial distribution of correlation coefficient between NDVI and MMCP_d_ in China from 1991 to 2020. Each color indicates a range of correlation coefficient between NDVI and MMCP_d_.

Analysis of the NDVI and multiyear average maximum consecutive precipitation days ($MMCP_{d}$) identified strong positive correlations in the Southwestern TG regions, Songnen Plain, and Eastern Inner Mongolia Plateau (TD, TG) from 1991 to 2020 (Figure 12). Extremely strong negative correlations (−1.0 to −0.6) dominated the SE regions encircled by the Hengduan, Daba, Wu, and Xuefeng Mountains during the study period. Complex topography, steep slopes, and low water retention soils in these areas exacerbate soil erosion and vegetation loss during prolonged rainfall. Negative correlations were also observed in the Tianshan, Kunlun, and Qilian Mountains (TD), the Yangtze River Delta (SE), and the Qiongzhou Strait (TM) from 1991 to 2020.

#### 3.4.4. Impact of Multiyear Average Precipitation Amount (MP_a_) on Vegetation Coverage

**Figure 13:**
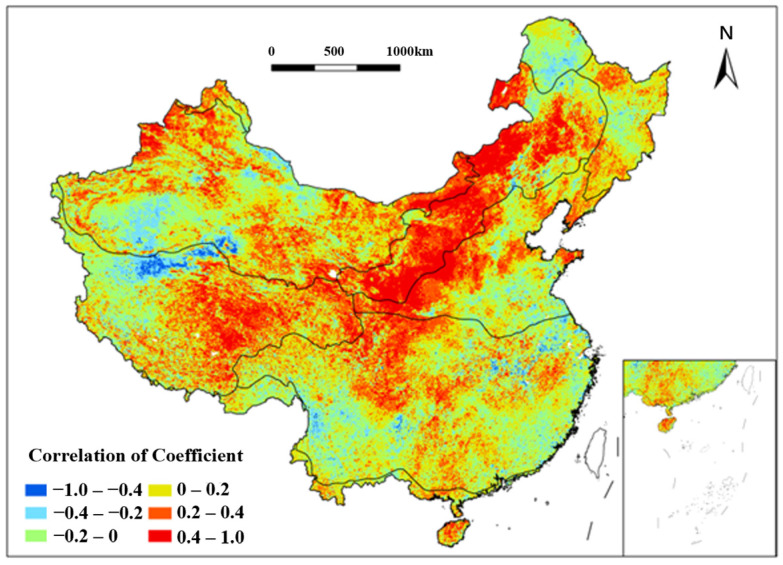
Spatial distribution of correlation coefficient between NDVI and MP_a_ in China from 1991 to 2020. Each color indicates a range of correlation coefficient between NDVI and MP_a_.

Analysis of the NDVI and multiyear average precipitation amount ($MP_{a}$) demonstrated extensive strong positive correlations (0.6–1.0) north of the Qinling–Huaihe Line, particularly in the Loess Plateau (TG, TM, SE) and Greater Khingan Mountains (TG, TD) from 1991 to 2020 (Figure 13). These temperate regions benefit from distinct wet–dry seasons, where synchronized rain and heat enhance vegetation growth. Positive correlations also occurred west of the Hengduan Mountains (AV), the Tianshan–Qilian Mountains (TD), and the Yangtze River mid-lower reaches (SE) during the study period. Conversely, strong negative correlations were observed south of the Kunlun Mountains and Tarim Basin from 1991 to 2020. Overall, $MP_{a}$ positively influences vegetation coverage.

#### 3.4.5. Impact of Annual Mean Consecutive Precipitation Amount (MACP_a_) on Vegetation Coverage

**Figure 14:**
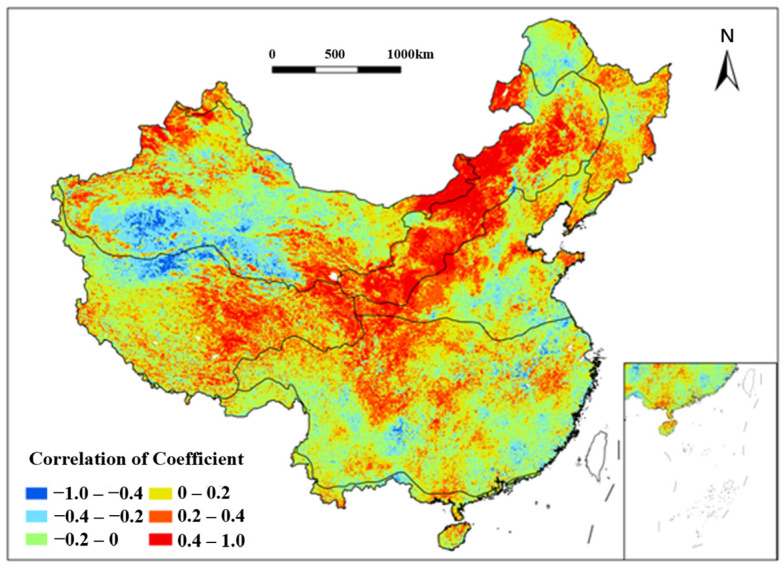
Spatial distribution of correlation coefficient between NDVI and MACP_a_ in China from 1991 to 2020. Each color indicates a range of correlation coefficient between NDVI and MACP_a_.

A correlation analysis between the NDVI and mean annual consecutive precipitation amount ($MACP_{a}$) revealed widespread strong positive correlations across the TG region from 1991 to 2020 (Figure 14). Extremely strong positive correlations (0.6–1.0) were concentrated in the Eastern Inner Mongolia Plateau, the Central and Northwestern Greater Khingan Mountains, and the Helan, Yin, Taihang, and Loess Plateau regions (AV, TG, WT, SE) during the study period. These patterns may be attributed to the complex vegetation structure and high biodiversity in TG regions, where increased precipitation enhances vegetation productivity. Strong positive correlations were also observed in Northern Tianshan Mountains (TD) and Eastern Tibetan Plateau (AV) regions from 1991 to 2020. In contrast, significant negative correlations were identified on both sides of the Kunlun Mountains (AV, TD) during the study period, likely due to limited marine moisture transport, low precipitation, and harsh alpine climates, which constrain vegetation growth. Overall, $MACP_{a}$ exerts a positive influence on vegetation.

#### 3.4.6. Impact of Multiyear Average Consecutive Precipitation Days (MCP_d_) on Vegetation Coverage

**Figure 15:**
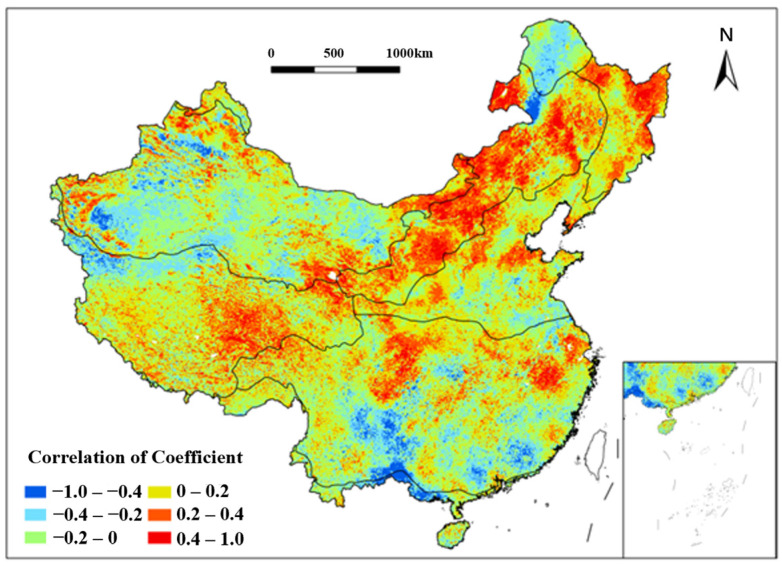
Spatial distribution of correlation coefficient between NDVI and MCP_d_ in China from 1991 to 2020. Each color indicates a range of correlation coefficient between NDVI and MCP_d_.

The research indicates that an increase in multiyear mean of continuous precipitation days ($MCP_{d}$) exerts a positive influence on vegetation cover across most of the TG region, with the effect centered over the Yin Mountains and the Loess Plateau and diminishing radially outward (Figure 15). In the TC region, $MCP_{d}$ shows a relatively strong correlation with local NDVI in the area where the southern Lesser Khingan Mountains meet the Changbai Mountains, whereas an opposite pattern is observed in the region where the northern Lesser Khingan Mountains adjoin the Greater Khingan Mountains. Consequently, vegetation in the CT region is generally negatively correlated with $MCP_{d}$. A similar negative correlation is also found along the transitional zone between the southern Yunnan–Guizhou Plateau and the TM region. Overall, the impact of $MCP_{d}$ on forest and grassland ecosystems is comparable to that of $MACP_{a}$, although its effect is slightly weaker in certain regions and exhibits markedly negative influences on vegetation growth in localized areas.

#### 3.4.7. Multiple Linear Regression Analysis of Precipitation Factors and Vegetation Coverage

The correlation coefficients derived from the multiple linear regression analysis are presented in Table 2. The results indicate that among the precipitation metrics examined, $MP_{a}$, $MACP_{a}$, and $MCP_{d}$ exerted the most substantial influences on vegetation growth across China. In contrast, $MACP_{t}$ yielded meaningful results only in the TC region during the 2011–2020 period; therefore, it was not explored further in other regions.

In the CT region, the effects of precipitation factors were primarily concentrated between 1991 and 2010, with $MCP_{d}$ exhibiting the strongest influence and maintaining a significant negative correlation with NDVI throughout this 20-year period. Additionally, during 2001–2010, both $MP_{a}$ and $MACP_{a}$ also had significant negative impacts on local vegetation growth, with correlation coefficients of −0.554 and −0.558, respectively.

The precipitation factors studied showed notable effects on NDVI in the WT and AV regions only during 1991–2000. In the WT region, only $MACP_{a}$ displayed a significant positive correlation (coefficient = 0.609), whereas in the AV region, both $MMCP_{d}$ and $MP_{a}$ showed significant positive correlations (coefficients = 0.608 and 0.780, respectively).

As described in Section 3.1 and Section 3.2, all precipitation metrics were generally higher in the TM region and relatively lower in the TG region. Nevertheless, according to Table 2, the NDVI in both regions responded significantly to multiple precipitation factors; specifically, $MMCP_{d}$ and $MACP_{a}$ had significant positive effects on TM over the entire 20-year span. In the TG region, $MMCP_{a}$, $MP_{a}$, and $MACP_{a}$ all maintained significant positive correlations with NDVI during 2001–2020.

The TD region, being the most arid, exhibited relatively weak and discontinuous responses to precipitation factors. However, during 2011–2020, a strong negative correlation emerged between the NDVI and $MCP_{d}$ (−0.892), the strongest negative relationship observed in this study, second in magnitude only to the strong positive correlation between NDVI in TG and $MACP_{a}$ (0.893) during the same period.

In the TC region, NDVI was noticeably influenced by precipitation factors during 2011–2020, with $MMCP_{d}$ showing the strongest negative correlation (coefficient = −0.784). In the SE region, significant positive correlations were observed with $MP_{a}$ and $MACP_{a}$ during 1991–2000, with coefficients of 0.695 and 0.630, respectively, indicating comparable magnitudes of effect.

## 4. Discussion

Consistent with the prevailing consensus in the scientific community, the findings of this study confirm the significant influence of precipitation on plant growth and its critical role in the recovery of degraded grassland and forest ecosystems [69,70].

While previous research on precipitation effects on vegetation cover has largely concentrated on “annual precipitation” or “extreme precipitation events,” the present study systematically introduces a new indicator system for “continuous precipitation events.” This system, based on single-event rainfall amount, duration, and inter-event frequency, includes $MACP_{a}$, $MMCP_{a}$, $MCP_{d}$, $MMCP_{d}$, and $MACP_{t}$. For example, Chen et al. demonstrated that vegetation in semi-arid regions like Inner Mongolia was most responsive to the precipitation amount [71,72]. Our results further indicate that this responsiveness is predominantly governed by $MACP_{a}$, with its influence varying over time. Vegetation preferences for particular precipitation event characteristics may shift with time, location, and vegetation status. Therefore, a multidimensional approach—encompassing single-event amount, duration, and frequency—yields more specific and robust findings than a single focus on total precipitation, particularly in the context of spatiotemporal dynamics. Such an approach is essential for water-limited ecosystems.

In the CT and TC regions, where the forest has been degraded due to tree logging, there is a reliance on short-duration, high-frequency rainfall events, which support seedling survival and growth in harvested areas. In logged areas, where vegetation is reduced to low-lying seedlings, prolonged rainfall increases susceptibility to debris flow and soil erosion, thereby endangering the persistence of these vulnerable plants. In contrast, the forest in the TM region, which has not been seriously harvested, benefits from longer-duration, higher-intensity precipitation events that enhance nutrient uptake by understory vegetation through canopy leaching. In the TG region dominated by grassland ecosystems, increments in single-event rainfall amounts promote the growth of low-stature herbaceous plants. Notably, in the severely degraded southwestern grasslands, extended rainfall duration also contributed positively to grassland restoration. Driven by the flat topography and arid climate, appropriate precipitation increases in this region promote both plant carbon assimilation and soil microbial vitality, effectively supporting the restoration of degraded grassland ecosystems [73,74].

In certain regions, however, harsh climatic conditions or intensive anthropogenic disturbances are the dominant drivers of plant growth and distribution. For instance, in the high-altitude, low-pressure AV region and the arid TD region, plants have evolved specialized structural adaptations that reduce their reliance on water availability, resulting in weak or even negative correlations with various precipitation indicators [75,76]. In the densely populated and highly developed SE and WT regions, NDVI increases since the turn of the 21st century have shown little responsiveness to precipitation events. Nevertheless, during the 1990–2000 period, plant growth in these two regions still exhibited a positive response to increases in single-event rainfall amount. NDVI growth during 1991–2000 was closely associated with increased single-event rainfall, whereas this linkage was no longer evident in 2001–2020. This shift is likely attributable to enhanced anthropogenic pressures—including urban expansion—which appear to have overridden natural precipitation controls on vegetation. Further research is needed to quantify the mechanisms and extent of human influence on regional plant growth. Beyond the SE and WT regions, human activities also play a significant role in driving vegetation cover change in other areas, notably afforestation and irrigation in the TD region. Therefore, the potential bias caused by human factors should be taken into account when interpreting the relationships between NDVI and precipitation across the different vegetation zones examined.

## 5. Conclusions

As an essential component of biogeochemical cycles, precipitation is intrinsically linked to plant growth and ecosystem recovery. Consequently, this study examines the impacts of various precipitation indicators on NDVI across different study regions. $MACP_{a}$ and $MMCP_{a}$ exhibited the highest values in the TM region, decreasing from south to north and east to west. A notable exception occurred in the TD region, where $MACP_{a}$ on the northwestern side was markedly higher than on the eastern side, with local areas approaching the level of the TG region. $MCP_{d}$ and $MMCP_{d}$ showed a west-high, east-low pattern in the SE region. $MACP_{t}$ peaked in the SE region and decreased north to south across the TD, WT, and TC regions.

From 1991 to 2000, NDVI showed overall recovery, but there was degradation in the eastern and western parts of the TG and WT regions. From 2001 to 2010, previously degraded areas recovered rapidly, while the TD, AV, SE, and Central TG regions experienced serious degradation. From 2011 to 2020, the TC and CT regions began to degrade and the WT region suffered unprecedented degradation, while most other regions exhibited large-scale recovery. Based on the correlation patterns between NDVI and continuous precipitation indices, the eight regions can be grouped into four response types:

The precipitation-sensitive type, which includes the TM, TG, and parts of the SE regions, where large-scale or frequent continuous precipitation events significantly promote vegetation recovery. The TM region benefits from prolonged duration, while the TG region responds more to single-event amount and frequency. The recovery-stage-sensitive type comprising the CT and TC regions, where these degraded forest regions at higher latitudes have increased event scales that tend to exert negative effects. In the SE and WT regions, which are classified as the human-dominated type, precipitation factors play a limited role due to intensive human activities (urbanization, land-use change). However, during 1991–2000, $MACP_{a}$ was a significant positive driver of NDVI in these regions, indicating that natural precipitation control has been progressively overridden. The adaptation-buffered type, which includes the AV and TD region, where vegetation in these geographically extreme regions has evolved specialized structural adaptations (e.g., leaf thickening, water storage tissues, carbon allocation strategies) that reduce reliance on water availability; consequently, most precipitation indicators showed weak or negative correlations.

Compared with traditional annual precipitation totals ($MP_{a}$), $MACP_{a}$ played a more significant role in most vegetation zones, particularly in areas with relatively low vegetation cover. $MMCP_{a}$ showed comparable influence to $MACP_{a}$. The proposed framework—integrating event amount, duration, and frequency with vegetation population and distribution characteristics—refines our understanding of how water availability specifically affects different regional and functional vegetation types. This refinement is essential for formulating and adjusting region-specific ecological conservation strategies.

## Figures and Tables

**Table 1:** National and regional values of precipitation indexes in China during 1991–2020.

Vegetation Regions	MP_a_ (mm)	MCP_d_ (Days)	MMCP_d_ (Days)	MACP_a_ (mm)	MMCP_a_ (mm)	MACP_t_ (Times)
National (467)	823.5	2.3	9	16.7	132.8	57.7
CT (6)	488.3	2.1	8.5	8.7	80.9	56.7
TC (27)	657.3	2.0	8.0	11.0	97.6	59.4
TG (70)	353.6	1.7	5.8	8.1	61.6	43.7
TD (44)	123.2	1.5	4.4	3.8	24.0	29.8
WT (87)	623.3	1.8	6.1	14.0	112.2	44.4
AV (27)	450.4	2.6	12.0	9.7	74.9	45.9
SE (189)	1254.5	2.8	11.7	24.3	189.7	77.9
TM (17)	1778.4	3.2	15.0	37.1	346.0	48.5

Numbers following “Vegetation regions” represent the quantity of meteorological stations within each region.

**Table 2:** Multiple linear regression analysis of precipitation factors and vegetation coverage during 1991–2020.

Influencing Factors	CT	WT	AV	TM	TG	TD
1991–2000						
MMCPa	0.192	0.322	0.608 *	0.420	0.524	−0.300
MACPt	0.463	0.021	0.078	−0.310	−0.157	0.289
MMCPd	−0.295	−0.145	0.353	0.631 *	0.320	−0.560 *
MPa	−0.395	0.539	0.780 **	0.491	0.335	0.449
MACPa	−0.497	0.609 *	0.487	0.681 *	0.394	−0.394
MCPd	−0.727 **	−0.198	0.454	0.603 *	−0.043	−0.455
2001–2010						
MMCPa	0.073	0.181	−0.455	0.214	0.618 *	0.107
MACPt	0.153	0.295	0.362	0.134	0.110	0.299
MMCPd	−0.264	−0.153	−0.251	0.585 *	0.067	−0.217
MPa	−0.554 *	0.310	0.091	0.497	0.599 *	−0.071
MACPa	−0.595 *	0.199	−0.274	0.466	0.648 *	−0.022
MCPd	−0.558 *	−0.277	−0.311	0.399	0.615 *	0.014
2011–2020						
MMCPa	0.076	−0.211	0.126	0.114	0.550 *	0.042
MACPt	0.297	−0.072	0.115	0.112	0.157	0.218
MMCPd	0.133	−0.101	0.176	0.471	0.210	−0.216
MPa	0.134	−0.144	0.331	0.765 **	0.837 **	0.239
MACPa	−0.040	−0.068	0.106	0.704 *	0.893 **	−0.243
MCPd	−0.067	−0.305	0.078	0.397	0.259	−0.892 **

* *p* < 0.05; ** *p* < 0.01.

## Data Availability

China Meteorological Data Network (http://data.cma.cn (accessed on 7 August 2022)); Geographic remote sensing ecological network platform (www.gisrs.cn (accessed on 24 October 2024)).

## Abbreviations

AP_a_	Annual precipitation amount (mm)
MP_a_	Multiyear mean of annual precipitation amount (mm)
CP	Continuous precipitation (an event of precipitation every day during a period of time)
CP_d_	Continuous precipitation days (days in one event of continuous precipitation)
ACP_d_	Annual mean of continuous precipitation days (days)
MCP_d_	Multiyear mean of continuous precipitation days (days)
AMCP_d_	Annual maximum of continuous precipitation days (days)
MMCP_d_	Multiyear mean of annual maximum of continuous precipitation days (days)
CP_a_	Continuous precipitation amount (amount of precipitation in one event of continuous precipitation)
ACP_a_	Annual mean of continuous precipitation amount (mm)
MACP_a_	Multiyear mean of continuous precipitation amount (mm)
AMCP_a_	Annual maximum of continuous precipitation amount (mm)
MMCP_a_	Multiyear mean of annual maximum of continuous precipitation amount (mm)
ACP_t_	Annual continuous precipitation times (times)
MACP_t_	Multiyear mean of annual continuous precipitation times (times)
MMCP_t_	Multiyear maximum of annual continuous precipitation times (times)
FCP	Frequency in different ranges of ACPa
MFCP	Frequency in different ranges of MACPa
